# Ultra Short Heart Rate Variability Predicts Clinical Outcomes in Patients with a Clinical Presentation Consistent with Myocarditis: A Derivation Cohort Analysis

**DOI:** 10.3390/jcm12010089

**Published:** 2022-12-22

**Authors:** Shay Perek, Udi Nussinovitch, Reut Cohen, Yori Gidron, Ayelet Raz-Pasteur

**Affiliations:** 1 Department of Internal Medicine A, Rambam Health Care Campus, Haifa 3109601, Israel; 2 Department of Emergency Medicine, Rambam Health Care Campus, Haifa 3109601, Israel; 3 The Ruth and Bruce Rappaport Faculty of Medicine, The Technion—Israel Institute of Technology, Haifa 3109601, Israel; 4 Department of Cardiology, Wolfson Medical Center, Holon 5822012, Israel; 5 Faculty of Medicine, Tel Aviv University, Tel Aviv 6997801, Israel; 6 Department of Nursing, Faculty of Social Welfare and Health Sciences, University of Haifa, Haifa 3498838, Israel

**Keywords:** myocarditis, prognosis, electrocardiogram, heart rate variability

## Abstract

Myocarditis prognosis varies substantially, hence identification of novel prognostic factors is crucial. The prognostic role of ultra-short heart-rate variability (HRV) in myocarditis remains unknown. In a retrospective study, adult patients admitted to a tertiary hospital due to clinically suspected myocarditis were included. Clinical, laboratory and HRV parameters were assessed as predictors of severe short term complications (heart failure (HF), dilated cardiomyopathy—DCM, ventricular arrhythmia—VA and death), utilizing logistic regression (LR). Accuracy was evaluated with receiver operating characteristic (ROC) curve area under the curve (AUC). HRV indices included standard deviation of normal beat intervals (SDNN) and root mean square of successive differences (RMSSD). 115 patients, aged 34 (±13) years old, were examined. Six patients (5%) developed severe HFrEF. RMSSD was included in a multivariate LR model (RMSSD < 10.72 ms adjusted odds ratio (AOR) 14.056, *p*-value 0.024). Model classification accuracy was very good, with an AUC of 86%. Eight patients (7%) developed DCM. RMSSD < 10.72 ms was included in a multivariate classification model (AOR 8.826, *p*-value 0.013); model classification AUC of 82%. HRV did not predict development of VA or death. SDNN and especially RMSSD may be prognostic indicators in myocarditis.

## 1. Introduction

Myocarditis is an inflammatory disease of the myocardium. In the majority of cases, myocarditis results from common viral infections, including COVID-19 [1]. Patients present with a wide range of symptoms, from fatigue, mild dyspnea or chest pain to cardiogenic shock, arrhythmia and death [2]. Therefore, early risk stratification is critical for acute myocarditis treatment and management. Current predictors of adverse disease course include clinical parameters (e.g., low systolic blood pressure (SBP), [3]), Electrocardiogram (ECG) abnormalities (e.g., long QRS interval [3,4], widened QRS-T angle [5]) and echocardiographic or cardiac magnetic resonance imaging findings (e.g., reduced left ventricular ejection fraction (LVEF) [4,6], and late gadolinium enhancement [7]). However, some of these processes (e.g., inflammation) and prognostic factors (e.g., SBP) are associated with and are modulated by the nervous system, particularly by the vagal nerve. The vagus inhibits blood pressure via the barroreflex and inhibits inflammation via two routes. First, by activating the hypothalamic-pituitary-adrenal axis resulting in cortisol suppressing inflammation. Second, through the descending vagal branch converting to a sympathetic branch that innervates the spleen, where responding T-cells secrete acetylcholine to suppress cytokine synthesis from splenic macrophages [8]. Heart rate variability (HRV) is the fluctuation in the time intervals between adjacent heartbeats. HRV indexes neuro-cardiac function and is generated by heart-brain interactions and dynamic non-linear autonomic nervous system processes [9]. HRV is strongly correlated with actual activity in the vagal nerve [10]. Several studies have demonstrated good correlation between specific ultra-short HRV (less than 5 minutes) indices and longer HRV recording [11,12,13,14,15]. HRV research has traditionally focused on linear time-domain variables such as the Standard Deviation of all normal to normal RR [NN] intervals (SDNN) and the Root Mean Square of Successive Differences between adjacent NN intervals (RMSSD) [16].

Reduced HRV as recorded from admission and discharge 10-second ECGs, has been found to be a significant and independent predictor of all-cause mortality in patients with ST elevation myocardial infarction [17]. This was also demonstrated in a meta-analysis of 21 studies showing that high HRV predicts on average 4 times a chance to survive after MI [18]. Higher SDNN in patients with COVID-19 has been shown to predict greater chance of survival, while low SDNN was found to be associated with early intensive care unit admission [19]. Furthermore, HRV has been established as a tool for rapid risk stratification in the Emergency Department (ED), in patients with malignancy [20]. Vagal nerve activity, quantified with HRV, has also been shown to predict survival in pancreatic cancer patients [21]. HRV time-domain indicators, including SDNN and RMSSD, of children diagnosed with viral myocarditis and presenting with ventricular arrhythmia (VA), were significantly lower compared to pediatric patients with myocarditis in which arrhythmias were not recorded [22]. Yet, studies which assess the prognostic value of HRV indices, measured during very short periods, in non-pediatric patients with myocarditis are lacking. Therefore, the aim of this study was to investigate the short and long term prognostic significance of HRV parameters in adults with myocarditis.

## 2. Materials and Methods

### 2.1. Study Design and Population

A retrospective single center analysis, based on the Rambam health care campus (RHCC; Haifa, Israel) database, of all hospital admissions, from January 2010 to June 2015, was conducted. Patients arriving at RHCC after June 2015 were not included, due to the use of a different ECG acquisition and coding system. Patients aged 18 years and older, who presented to the ED with clinically suspected new-onset myocarditis, based on the 2013 European society of cardiology position statement on myocardial and pericardial disease [23], were considered. Briefly, diagnosis of clinically suspected myocarditis was established based on at least one clinical and one diagnostic criteria (electrocardiographic, biochemical, or imaging), in the absence of angiographically detectable coronary artery disease (e.g., coronary stenosis ≥50%, when such an involvement was suspected), known pre-existing cardiovascular disease or extra-cardiac causes, which could explain the syndrome. Myocarditis is characterized by an abnormal 12-lead ECG, a Holter test, a stress test, or elevated serum Troponin levels. Alternatively, there is evidence of edema and/or late gadolinium enhancement of classical myocarditic pattern on cardiovascular magnetic resonance imaging, or other imaging findings suggestive of functional and structural abnormalities in LV or RV.

Based on the duration of the symptoms (days up to 3 months and >3 months, respectively), patients were classified as having suspected acute or subacute/chronic myocarditis.

Excluded were patients who did not have an ECG record from their ED visit, as well as recordings with ectopic cardiac activity (any non-sinus rhythm including atrial fibrillation or flutter, premature beats), as those might significantly affect computation of HRV parameters. Additionally, patients with low resolution ECG recordings were excluded. Furthermore, chronic treatment with beta or calcium channel blockers, resulted in exclusion. The institutional review board (IRB) approved this study (approval number 0603-16-RMB). Since all the data was retrospectively collected, individual informed consent was waived by the IRB.

### 2.2. Data Collection

All ED visits and discharge letters from the study period, were screened for a diagnosis of myocarditis, utilizing a designated computer software (MDClone, Beer-Sheva, Israel). Potential eligible patients’ electronic medical records (EMRs) were reviewed to verify eligibility. Medical history, presenting symptoms, ED vital signs and laboratory results, including complete blood count, chemistry panel and troponin levels were measured. In addition, echocardiography (echo) and magnetic resonance imaging (MRI) results were considered. Myocarditis workup outcome, disease-specific complications during index hospitalization (e.g., heart failure and arrhythmia) and date of death, were collected with the MDClone software, or by reviewing patients’ EMR. ECGs were recorded with ECG LAN mobile wireless systems (Norav Medical, Yokneam, Israel).

### 2.3. ECG and HRV Analysis

Patients arrived at RHCC ED and underwent a 10-second resting ECG LAN Green—mobile wireless model (Norav Medical, Yokneam, Israel), while lying motionless in a supine position for at least 30 seconds. The ECG electrodes were placed in anatomical positions according to standard procedure using a precordial ECG lead positioning system (Tapuz Medical, Caesarea, Israel). Resting ECG files were visualized with a viewing software (Resting ECG version 5.62 (Norav Medical, Yokneam, Israel)), analyzed with a custom version of the HRV analysis software able to import 10-second recordings (HRV version 5.62 (Norav Medical, Yokneam, Israel)). Ultra short HRV parameters, were computed automatically, utilizing this software. Additionally, ECGs were manually checked and recordings with disturbances, which could potentially affect accurate measurement of HRV, such as excessive noise, low resolution, and sudden baseline instability or spikes, were excluded from the analysis. ECGs which contained premature ventricular beats, premature supraventricular beats, atrial fibrillation and second or third degree atrioventricular block were excluded as well. This study focused on time-domain variables (including SDNN and RMSSD).

### 2.4. Endpoints

Outcomes included development of new heart failure with reduced ejection fraction (HFrEF), dilated cardiomyopathy (DCM; defined in this study as dilation and impaired contraction of one or both ventricles, in the absence of another cardiovascular condition sufficient to explain the observed myocardial abnormality (e.g., hypertension, valvular disease or ischemic heart disease)) and VA (including ventricular tachycardia (VT) or fibrillation (VF))—all documented during the index hospitalization. The severity of HFrEF was determined based on echocardiographic findings. LVEF under 50% was considered mild HFrEF, LVEF under 40%—moderate HFrEF and LVEF of 30% or less—wad regarded as severe HFrEF. This scale has been used at RHCC for echocardiographic assessment of heart failure severity. Additionally, a composite outcome, which was comprised of in-hospital mortality (during index hospitalization), VA and severe HFrEF was computed. Survival analysis was also carried out.

### 2.5. Statistical Analysis

The study database was analyzed and artwork created with R software (version 4.0.3, The R Foundation for Statistical Computing, Vienna, Austria). Descriptive statistics is presented with means (±standard deviation) or number (with percentage). HRV indices for each patient in the study cohort, were compared with published normal value ranges, corrected for age and gender [24]. Correlations between variables and Boolean outcomes were tested with univariate logistic regression (LR) and presented as odds ratio (OR) with *p*-values. Variables found to have statistical significance (*p*-value < 0.05) or trend (*p*-value < 0.09) in univariate analysis, were introduced into a multivariate LR model, in a backward stepwise fashion. Variables with significant multivariate LR correlations (*p*-value < 0.05) are presented with adjusted OR (AOR) as well as 95% confidence intervals (CI) and *p*-values. Multivariate model classification accuracy is specified with receiver operating characteristic (ROC) curves, including the area under the curve (AUC), with 95% CIs based on bootstrapping methodology, as well as Hosmer and Lemeshow goodness-of-fit (HLGOF) *p*-value and overall model *p*-value. In addition, multivariate random forest (RF) classification was carried out for Boolean outcomes. Variable significance in multivariate RF models, is presented as mean decrease in model accuracy and mean decrease in Gini (i.e., how each variable contributes to the homogeneity of the nodes and leaves in the model), while classifier accuracy is displayed as ROC AUC and out-of-bag (OOB) estimate of error rate. Survival analysis was performed with Cox regression. HRV parameters were examined as both continuous and dichotomous variables (e.g., smaller than quartile 1 (Q1; 25th percentile)).

## 3. Results

### 3.1. Patient Characteristics

One-hundred and seventy patients were initially considered for this analysis. Of them, 8 patients were excluded as they were receiving beta or calcium channel blockers during ECG acquisition in the ED. An additional 11 did not have digital ECG files, while 24 were excluded due to low technical ECG quality. Finally, 12 patients presented with irregular heartbeats documented on ECG. The final study group included 115 patients, aged 34 (±13) years old. All patients reported new symptoms or worsening symptoms within 3 months prior to admission (i.e., dyspnea at rest or during exercise, as well as fatigue), which allowed categorization as ‘suspected acute myocarditis’. The majority (102, 89%) were men. With regard to patient inclusion based on the criteria for clinically suspected myocarditis, all 115 patients presented to the ED with acute chest pain, 114 (99%) had elevated troponin levels and 58 (50%) were found to have myocarditis-specific MRI or echocardiography findings. Cardiac catheterization was performed in 27 (23%) of patients, when ischemic heart disease was suspected as an alternative diagnosis. None of the included patients had unexplained cardiogenic shock or required endomyocardial biopsy. Patient clinical, laboratory and electrocardiographic characteristics, along with HRV indices and imaging features, are detailed in Table 1.

Notably, HRV parameters were relatively low in the study cohort. Compared with published normal 10-second HRV values corrected for age and gender, [24] SDNN (median 23.0 ms, inter-quartile range 11.25–35.44 ms) was found to be lower than median values in 94 (82%) patients, with 26 (23%) patients having SDNN values lower than the 2nd percentile of their age and gender corrected range. As for RMSSD (median 21.5 ms, inter-quartile range 10.72–34.65 ms), 93 (81%) patients and 33 (29%) patients had values lower than age and gender corrected median and 2nd percentile, respectively.

### 3.2. Heart Failure

Eight patients (7% (95% CI 3–14%)) developed new HFrEF of at least moderate degree, while 6 (5% (95% CI 2–11%)) of these patients were diagnosed with severe HFrEF. None of the patients developed cardiogenic shock or required hemodynamic support. For moderate HFrEF, 4 parameters were found to have univariate LR significant or trend correlations: age (OR 1.044 (95% CI 0.998–1.092), *p*-value 0.057), MAP (OR 1.081 (95% CI 1.026–1.139), *p*-value 0.003), SDNN < 11.25 ms (OR 5.763 (95% CI 1.283–25.876), *p*-value 0.022) and RMSSD < 10.72 ms (OR 10.956 (95% CI 2.071–57.937), *p*-value 0.004). Multivariate LR classification model included both MAP (AOR 1.074 (95% CI 1.014–1.137), *p*-value 0.014) and RMSSD < 10.72 ms (AOR 8.848 (95% CI 1.542–50.752), *p*-value 0.014); HLGOF *p*-value 0.703, overall model *p*-value 0.0002. Model classification was very good, with an AUC of 84% (95% CI 68–100%).

**Figure 1:**
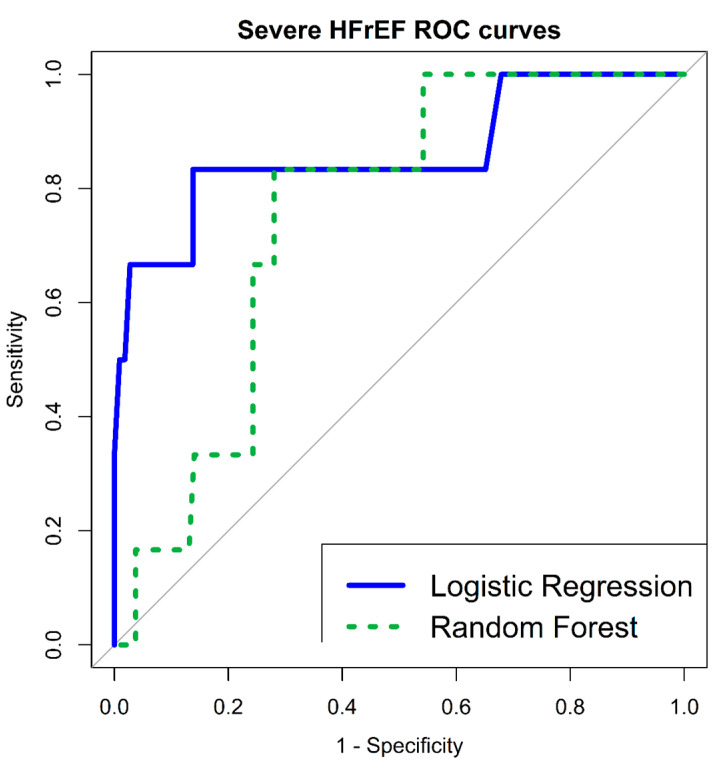
ROC curves—Severe HFrEF—multivariate LR and RF classification models. HFrEF—Heart Failure reduced Ejection Fraction; LR—Logistic Regression; RF—Random Forest; ROC—Receiver Operating Characteristic.

As for new severe HFrEF, Table 2 details both univariate LR correlations as well as RF multivariate variable importance. HRV parameters were found to have significant univariate LR correlations (SDNN < 11.25 ms (OR 6.719 (95% CI 1.161–38.869), *p*-value 0.033); RMSSD < 10.72 ms (OR 17.708 (95% CI 1.973–158.910), *p*-value 0.010), as well as significant RF multivariate importance (SDNN—mean decrease accuracy 5.216; RMSSD—mean decrease accuracy 1.722). Multivariate LR model included, again, MAP and RMSSD < 10.72 ms (AOR 1.090 (95% CI 1.018–1.167), *p*-value 0.012; AOR 14.056 (95% CI 1.400–141.093), *p*-value 0.024; respectively); HLGOF *p*-value 0.222, overall model *p*-value 0.0002. Multivariate LR model classification accuracy was very good, with an AUC of 86% (95% CI 65–100%). In addition, RF multivariate classifier AUC was 75% (95% CI 60–90%), with an OOB estimate of error rate—5.31%; as detailed in Figure 1.

### 3.3. Dilated Cardiomyopathy

Eight patients (7% (95% CI 3–14%)) developed DCM after suffering from myocarditis. 5 parameters were found to have univariate LR significant or trend correlations: age (OR 1.045 (95% CI 0.999–1.093), *p*-value 0.051), MAP (OR 1.071 (95% CI 1.019–1.127), *p*-value 0.007), platelets level (OR 1.006 (95% CI 0.998–1.013), *p*-value 0.092), as well as HRV indices—SDNN < 11.25 ms (OR 5.763 (95% CI 1.283–25.876), *p*-value 0.022) and RMSSD < 10.72 ms (OR 10.956 (95% CI 2.071–57.937), *p*-value 0.004).

**Figure 2:**
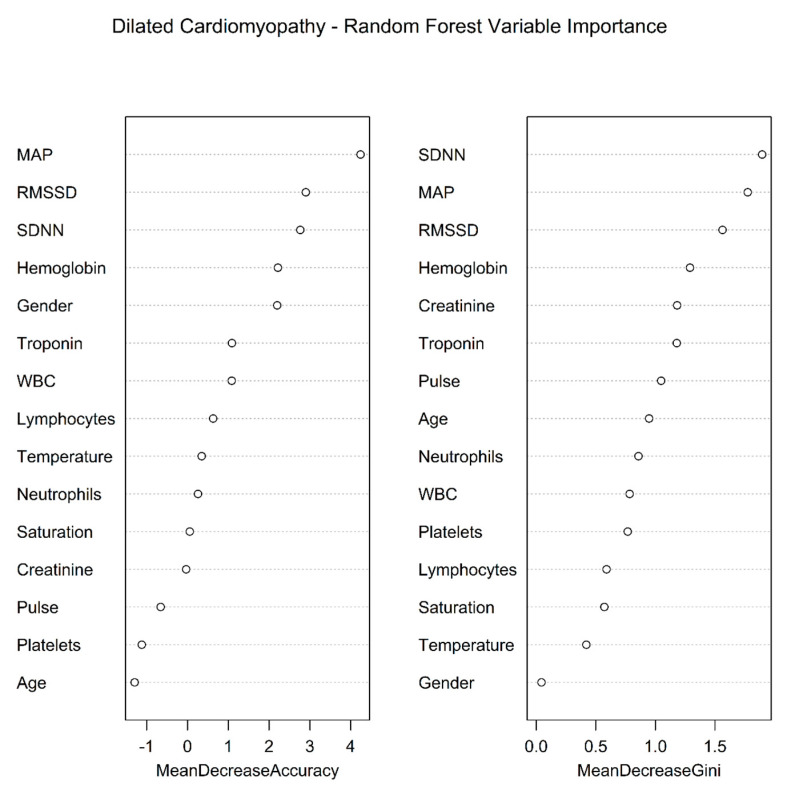
Dilated cardiomyopathy—RF variable importance. MAP—Mean Arterial Pressure; RF—Random Forest; RMSSD—Root Mean Square of Successive RR interval Differences; SDNN—Standard Deviation of NN intervals; WBC—White Blood Cells.

SDNN and RMSSD (along with MAP) were found to be of very significant importance in a RF multivariate classifier (Figure 2).

Multivariate LR classification model included both MAP (AOR 1.062 (95% CI 1.006–1.122), *p*-value 0.028) and RMSSD < 10.72 ms (AOR 8.826 (95% CI 1.576–49.430), *p*-value 0.013); HLGOF *p*-value 0.018, overall model *p*-value 0.0006. Model classification was very good, with an AUC of 82% (95% CI 62–100%). In addition, RF multivariate classifier AUC was 67% (95% CI 48–87%), with an OOB estimate of error rate—7.96%.

### 3.4. Ventricular Arrhythmia

Five patients (4% (95% CI 1–10%)) developed a VA during their index hospitalization (4 patients—non-sustained VT and 1 patient—VF). Ultra-short HRV indices were not found to predict the development of a VA: SDNN (OR 0.948 (95% CI 0.870–1.031), *p*-value 0.217), RMSSD (OR 0.972 (95% CI 0.912–1.036), *p*-value 0.395).

### 3.5. Mortality

With regard to all-cause mortality, 1 patient died during the index hospitalization, while an additional 5 patients died within the study follow-up period (median 8.6 years, Inter Quartile Range 7.2–9.8 years). Survival analysis with Cox regression demonstrated a non-significant association between HRV indices and mortality over time: SDNN—beta −0.02, HR 0.98, *p*-value 0.44 L RMSSD—beta −0.008, HR 0.99, *p*-value 0.64.

### 3.6. Severe Short-Term Disease-Specific Complications

**Figure 3:**
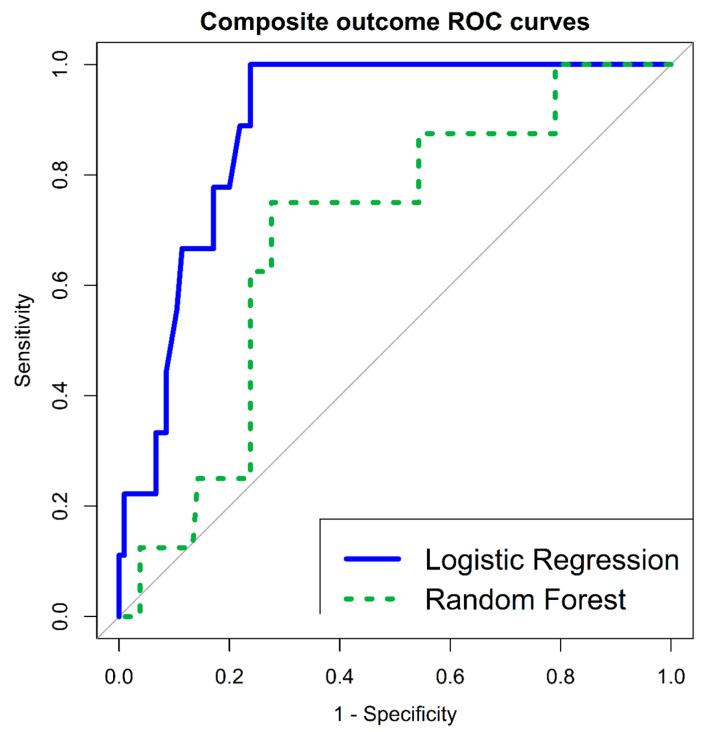
ROC curves—composite outcome (in-hospital mortality/VA/severe new HFrEF)—multivariate LR and RF classification models. HFrEF—Heart Failure reduced Ejection Fraction; LR—Logistic Regression; RF—Random Forest; ROC—Receiver Operating Characteristic; VA—Ventricular Arrhythmia.

Nine patients (8% (95% CI 4–15%)) were positive for a composite outcome of severe short term disease specific complications (including in-hospital mortality (during index hospitalization), VA and severe HFrEF). HRV indices were found to have both significant univariate LR correlations (SDNN < 11.25 ms (OR 4.270 (95% CI 1.062–17.166), *p*-value 0.040); RMSSD < 10.72 ms (OR 7.217 (95% CI 1.674–31.103), *p*-value 0.008), as well as very significant multivariate RF variable importance (SDNN—mean decrease accuracy 5.267; RMSSD—mean decrease accuracy 4.403). Multivariate LR classification model included 3 parameters: male gender (AOR 0.114 (95% CI 0.018–0.731), *p*-value 0.021); lymphocyte levels (AOR 0.211 (95% CI 0.046–0.973), *p*-value 0.046) and RMSSD < 10.72 ms (AOR 7.561 (95% CI 1.538–37.171), *p*-value 0.012); HLGOF *p*-value 0.161, overall model *p*-value 0.0007. Model classification was very good, with an AUC of 89% (95% CI 82–96%). In addition, RF multivariate classifier AUC was 69% (95% CI 51–87%), with an OOB estimate of error rate—7.08%; as detailed in Table 3 and Figure 3.

## 4. Discussion

The broad clinical presentation of myocarditis, from non-specific symptoms, to heart failure and fulminant hemodynamic collapse, underscores the need for early, reliable predictors of outcomes. While cardiac MRI, right heart catheterization, serologic biomarkers and histopathologic characteristics have been found to predict the prognosis of acute myocarditis [25,26,27,28], identifying simple, readily available complementary tests, which can be carried out at the early stages of the disease, may be more practical means of risk stratification. Electrocardiographic diagnostic markers, including QRS segment fragmentation, have been described as possible simple bedside tools, for myocarditis diagnosis [29] and disease severity assessment [30]. In this retrospective (pseudo-prospective) analysis, we demonstrated for the first time, the use of ultra-short HRV indices, for prognostic purposes in suspected myocarditis. Both SDNN and especially RMSSD, were established as strong parameters for risk stratification, with regard to early short term serious adverse events. In fact, HRV indices importance, in both logistic regression analysis (univariate and multivariate) as well as random forest multivariate classification, strongly exceeded that of other common prognostic factors.

The RMSSD is the primary time-domain measure used to estimate the vagally mediated changes reflected in HRV [31]. In our study, we demonstrated the relation between low ED RMSSD in suspected myocarditis patients, and the development of new HFrEF and DCM. Importantly, resting heart rate, also influenced by vagal tone, did not show similar correlations. Low RMSSD has been established as a negative prognostic index in several patient populations. Reduced RMSSD after acute myocardial infarction was found to be independently correlated with 1-year mortality [32]. Moreover, among patients referred to a rapid response team, significantly higher HRV (including RMSSD), was observed in patients who achieved physiological stability and did not require intensive care unit admission [33].

The SDNN, which is affected mainly by the parasympathetic nervous system (through respiration), has been considered the benchmark for medical risk stratification, especially with regard to cardiovascular disease [34,35]. With regard to our cohort, SDNN was found to be a significant parameter in both RF and LR classifications of suspected myocarditis disease-specific complications. These results are in line with a meta-analysis of 21 studies showing a robust association between high HRV and reduced risk of post-MI mortality [18]. In addition, low ultra-short SDNN has been found to be an independent risk factor for 2-year mortality in patients recovering from ST-elevation MI [17].

Concerning the mechanism, since inflammation plays a role in the etiology of myocarditis [36], and since the vagus reflexively reduces inflammation [8], vagal modulation of this factor could also partly explain our findings.

There were several potential limitations to our study. First, this study is a retrospective single center analysis. Second, the diagnosis of ‘suspected myocarditis’ was made based upon a combination of clinical, laboratory and echocardiographic findings. None of the patients underwent endomyocardial biopsy to confirm the diagnosis. The lack of histologic findings did not allow for assessment of myocarditis pathologic diagnosis and study outcomes. Third, as the study deals with ultra-short HRV, 12 patients were excluded from the analysis due to irregular heartbeats captured during the 10-second ECG. These irregular beats may represent a more serious myocardial condition; thus their exclusion may have led to a selection bias. Having said that, the focus on patients presenting to the ED without arrhythmia documentation, provides an important spotlight on potentially lower risk myocarditis patients (i.e., without arrhythmia upon diagnosis). Forth, our study did not include neither asymptomatic myocarditis patients nor fulminant myocarditis patients requiring hemodynamic support. Therefore, it is unknown whether our results pertain to these patient populations. Additionally, since the overall number of adverse events in the tested population was low, the results of the multivariate logistic analysis should be interpreted with caution. Finally, the exact time patients laid down in a supine position prior to undergoing ECG is unknown. However, there is increasing evidence that even short stabilization periods prior to HRV measurement, is acceptable, especially in younger, healthier patients [37], and in static conditions [38].

## 5. Conclusions

Despite these limitations, this study clearly demonstrated that time domain ultra-short HRV indices based on ECG, carried out upon ED arrival, have the potential to serve as early risk stratification means for suspected myocarditis patients and predict crucial patient outcomes. Reduced RMSSD was correlated with short term severe disease sequela and their effect surpassed those of other known prognostic factors. Our findings should be validated, prior to implementation in patient management strategies. Furthermore, it is uncertain whether this tool can be used to identify patients who will respond to beta-blockers or to monitor their response.

## Figures and Tables

**Table 1:** Study population characteristics (*n* = 115).

	Mean (±Standard Deviation)/Number (Percentage of Group)
Age (years)	34 (±13)
Gender-Male	102 (89%)
Emergency Department Vital Signs	
MAP (mmHg)	93 (±14)
Pulse (BPM)	86 (±17)
Saturation (%)	98 (±2)
Temperature (°C)	37 (±1)
Emergency Department Labs	
WBC (10³/µL)	9.7 (±3.5)
Neutrophils (10³/µL)	6.5 (±3.3)
Lymphocytes (10³/µL)	1.8 (±0.7)
Hemoglobin (g/dL)	14.2 (±1.4)
Platelets (10³/µL)	219 (±73)
Troponin (ng/mL)	4.5 (±5.9)
Creatinine (mg/dL)	0.9 (±0.2)
Emergency Department ECG & HRV	
PR interval (ms)	149 (±24)
QRS interval (ms)	88 (±11)
QTC interval (ms; Bazzet)	403 (±32)
SDNN (ms)	30.1 (±33.7)
RMSSD (ms)	32.3 (±46.2)
MRI & Echo Findings	
Pericardial Effusion	10 (9%)
Wall Motion Abnormalities	31 (27%)
Late Gadolinium Enhancement	35 (30%)

BPM—Beats Per Minute; ECG—Electrocardiogram; MAP—Mean Arterial Pressure; RMSSD—Root Mean Square of Successive RR interval Differences; SDNN—Standard Deviation of NN intervals; HRV—Heart Rate Variability; WBC—White Blood Cells.

**Table 2:** New HFrEF of severe degree (per echo)—logistic regression and random forest classifiers.

	Logistic Regression—Univariate			Random Forest—Multivariate	
	OR	95% CI	*p*-Value	Mean Decrease Accuracy	Mean Decrease Gini
Age (years)	1.060	1.008–1.114	0.022 *	−0.776	0.982
Gender−Male	0.618	0.066–5.747	0.673	0.016	0.020
Emergency Department Vital Signs					
MAP (mmHg)	1.098	1.033–1.166	0.002 *	3.879	1.553
Pulse (BPM)	1.042	0.995–1.091	0.075	−1.628	0.782
Saturation (%)	0.717	0.477–1.078	0.111	−1.466	0.454
Temperature (°C)	1.316	0.390–4.439	0.657	−0.188	0.374
Emergency Department Labs					
WBC (10³/µL)	0.938	0.723–1.216	0.630	1.376	0.780
Neutrophils (10³/µL)	1.002	0.781–1.285	0.985	−0.024	0.522
Lymphocytes (10³/µL)	0.405	0.095–1.717	0.220	−1.133	0.837
Hemoglobin (g/dL)	0.559	0.311–1.003	0.051	0.559	0.942
Platelets (10³/µL)	1.005	0.997–1.013	0.181	−1.679	0.535
Troponin (ng/mL)	0.163	0.008–3.102	0.227	−0.434	0.846
Creatinine (mg/dL)	7.701	0.264–224.286	0.235	1.411	0.924
Emergency Department HRV					
SDNN (ms)	0.971	0.913–1.031	0.342	5.216	1.023
RMSSD (ms)	0.988	0.949–1.029	0.579	1.722	0.920
SDNN < 11.25 ms	6.719	1.161–38.869	0.033 *	/	/
RMSSD < 10.72 ms	17.708	1.973–158.910	0.010 *	/	/

* *p*-value < 0.05. BPM—Beats Per Minute; CI—Confidence Interval; HFrEF—Heart Failure reduced Ejection Fraction; MAP—Mean Arterial Pressure; OR—Odds Ratio; RMSSD—Root Mean Square of Successive RR interval Differences; SDNN—Standard Deviation of NN intervals; HRV—Heart Rate Variability; WBC—White Blood Cells.

**Table 3:** Composite outcome of severe short-term disease-specific complications (in-hospital mortality/VA/new severe HFrEF)—logistic regression and random forest classifiers.

	Logistic Regression—Univariate			Random Forest—Multivariate	
	OR	95% CI	*p*-Value	Mean Decrease Accuracy	Mean Decrease Gini
Age (years)	1.067	1.021–1.116	0.003 *	2.349	1.455
Gender-Male	0.208	0.045–0.963	0.044 *	0.267	0.092
Emergency Department Vital Signs					
MAP (mmHg)	1.057	1.009–1.108	0.018 *	1.859	1.945
Pulse (BPM)	1.036	0.996–1.077	0.071	0.172	0.776
Saturation (%)	0.806	0.557–1.167	0.254	−0.970	0.489
Temperature (°C)	1.549	0.571–4.201	0.389	0.345	0.514
Emergency Department Labs					
WBC (10³/µL)	1.016	0.842–1.226	0.860	2.269	0.999
Neutrophils (10³/µL)	1.154	0.985–1.353	0.075	1.489	0.955
Lymphocytes (10³/µL)	0.247	0.062–0.975	0.045 *	0.034	1.077
Hemoglobin (g/dL)	0.562	0.342–0.923	0.022 *	0.496	1.340
Platelets (10³/µL)	1.001	0.993–1.010	0.708	−0.404	0.912
Troponin (ng/mL)	0.802	0.605–1.061	0.124	−0.508	0.690
Creatinine (mg/dL)	2.152	0.088–52.634	0.638	0.986	0.974
Emergency Department HRV					
SDNN (ms)	0.967	0.918–1.019	0.215	5.267	1.225
RMSSD (ms)	0.989	0.958–1.021	0.524	4.403	1.363
SDNN < 11.25 ms	4.270	1.062–17.166	0.040 *	/	/
RMSSD < 10.72 ms	7.217	1.674–31.103	0.008 *	/	/

* *p*-value < 0.05. BPM—Beats Per Minute; CI—Confidence Interval; HFrEF—Heart Failure reduced Ejection Fraction; MAP—Mean Arterial Pressure; OR—Odds Ratio; RMSSD—Root Mean Square of Successive RR interval Differences; SDNN—Standard Deviation of NN intervals; HRV—Heart Rate Variability; WBC—White Blood Cells.

## Data Availability

The data presented in this study are openly available in FigShare at https://doi.org/10.6084/m9.figshare.21546969.v1, accessed on 12 November 2022.
